# *MYCN* Transgenic Zebrafish Model with the Characterization of Acute Myeloid Leukemia and Altered Hematopoiesis

**DOI:** 10.1371/journal.pone.0059070

**Published:** 2013-03-15

**Authors:** Li-Jing Shen, Fang-Yuan Chen, Yong Zhang, Lan-Fang Cao, Ying Kuang, Min Zhong, Ting Wang, Hua Zhong

**Affiliations:** 1 Department of Hematology, Renji Hospital, Shanghai Jiao Tong University School of Medicine, Shanghai, China; 2 Department of Pediatric, Renji Hospital, Shanghai Jiao Tong University School of Medicine, Shanghai, China; 3 Shanghai Research Center for Biomodel Organisms, Shanghai, China; B.C. Cancer Agency, Canada

## Abstract

**Background:**

Amplification of *MYCN* (N-Myc) oncogene has been reported as a frequent event and a poor prognostic marker in human acute myeloid leukemia (AML). The molecular mechanisms and transcriptional networks by which *MYCN* exerts its influence in AML are largely unknown.

**Methodology/Principal Findings:**

We introduced murine *MYCN* gene into embryonic zebrafish through a heat-shock promoter and established the stable germline Tg(*MYCN*:HSE:EGFP) zebrafish. N-Myc downstream regulated gene 1 (*NDRG1*), negatively controlled by *MYCN* in human and functionally involved in neutrophil maturation, was significantly under-expressed in this model. Using peripheral blood smear detection, histological section and flow cytometric analysis of single cell suspension from kidney and spleen, we found that *MYCN* overexpression promoted cell proliferation, enhanced the repopulating activity of myeloid cells and the accumulation of immature hematopoietic blast cells. *MYCN* enhanced primitive hematopoiesis by upregulating *scl* and *lmo2* expression and promoted myelopoiesis by inhibiting *gata1* expression and inducing *pu*.*1, mpo* expression. Microarray analysis identified that cell cycle, glycolysis/gluconeogenesis, MAPK/Ras, and p53-mediated apoptosis pathways were upregulated. In addition, mismatch repair, transforming and growth factor β (TGFβ) were downregulated in *MYCN*-overexpressing blood cells (*p*<0.01). All of these signaling pathways are critical in the proliferation and malignant transformation of blood cells.

**Conclusion/Significance:**

The above results induced by overexpression of *MYCN* closely resemble the main aspects of human AML, suggesting that *MYCN* plays a role in the etiology of AML. *MYCN* reprograms hematopoietic cell fate by regulating *NDRG1* and several lineage-specific hematopoietic transcription factors. Therefore, this *MYCN* transgenic zebrafish model facilitates dissection of *MYCN*-mediated signaling *in vivo*, and enables high-throughput scale screens to identify the potential therapeutic targets.

## Introduction

*Myc* was first discovered as the oncogene of avian leukemogenic retroviruses, and later found translocated in human lymphomas. *MYCN* (*N*-*Myc*) is one of the main members in *Myc* family which encodes MYC protein that forms heterodimer with MAX protein through their conserved basic helix-loop-helix/leucine zipper (bHLHZip) domains, which mediates DNA binding to cis-DNA sequences called E-box (CACGTG) in the promoter/enhancer regions of target genes. *MYCN* is expressed almost exclusively in embryonic tissues. Amplification of *MYCN* is frequently found in hematologic malignancies such as lymphoma and acute myeloid leukemia (AML), considered as a well-established poor prognostic marker in these diseases [1], [2], [3]. A recent quantitative real-time PCR (qRT-PCR) study on the CD34+ bone marrow cells collected from 37 AML patients revealed that 20% to 100% of the samples expressed 2 to 33-fold higher *MYCN* level than normal counterpart depending on the AML subtypes. The authors demonstrated that overexpression of *MYCN* rapidly caused acute myeloid leukemia in Mice [4]. However, the role of *MYCN* expression in the regulation of hematopoiesis and the mechanisms by which it acts to promote an aggressive maglinant phenotype remain largely unknown, and generation of transgenic offspring was not possible.

Heat shock protein (HSP) promoters have been extensively used in heterologous misexpression experimental systems for its highly conserved nature. The heat shock elements (HSE), short sequences present in all HSP promoters, have been identified to be essential for stress inducibility [5]. However, the *in vivo* application of this system in mammals is not applausible due to the strict control of the body temperature while others systems like insects and fish are ideal for the induction of a heat shock response at elevated temperatures[6]. The major problem observed in these experiments was high levels of background activity, while generation of transgenic lines can alleviate this problem, and meganuclease method leads to elevated integration efficiency of the DNA into the genome [7], thereby largely increasing the level of misexpressing cells and the number of transgenic offspring.

Zebrafish (Danio rerio) hematopoiesis shows anatomic, physiologic, and genetic conservation with that of humans, thus it has been used to study genetic pathways involved in human leukemia more recently [8]. Similar to mammal organisms, zebrafish experiences two waves (primitive and definitive) of hematopoiesis. Eventually by 4 days post fertilization (dpf), hematopoietic stem cells (HSCs) seed the kidney marrow, which is equivalent to the bone marrow in mammals [9]. Furthermore, ectopic expression of human or murine oncogenes driven by specific promoters in zebrafish has been shown to faithfully develop leukemias closely parallel to the human leukemia subtypes. Finally, the efficient reproduction and rapid development of the embryos of zebrafish allow it to become a convenient model to investigate the tumor development, and dissemination in real time, without having to sacrifice the animals.

In the present study, we established a stable line of zebrafish expressing the chimeric mouse *MYCN* and EGFP transgenes under control of a heat stress-inducible bidirectional promoter. This transgenic strategy is based on the *in vivo* leukemogenic effect of *AML1-ETO* driven by *hsp70* promoter in zebrafish [10] and overexpression *MYCN* induced AML in mice [4].

## Results

### Establishment of *MYCN* transgenic zebrafish line

**Figure 1:**
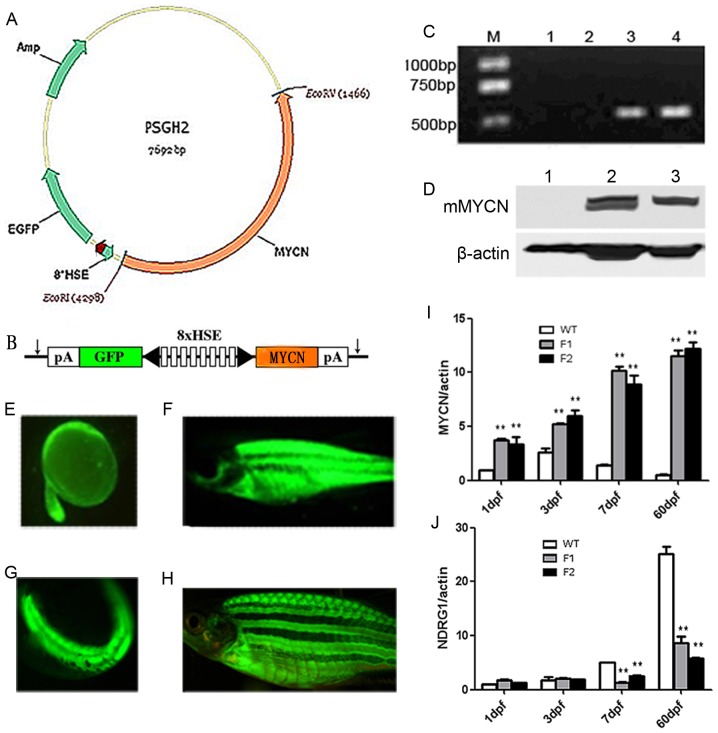
Generation of Tg(*MYCN*:HSE:EGFP) zebrafish line. (A) Schematic diagram of the structure of PSGH2/MYCN recombinant plasmid. A mouse-MYCN fragment was cloned from HA-MYCN plasmid and inserted into the EcoRI and EcoRV sites of the PSGH2 vector. (B) A schematic presentation of the heat shock element (HSE) promoter. The artificial promoter contains eight multimerized heat shock elements flanked by two minimal promoters in opposed orientation (black arrowhead). EGFP and *MYCN* are expressed from the bidirectional promoter. The vector is flanked by I-SceI meganuclease sites (arrows). pA, SV40 polyadenylation signal. (C) Transgenic verification by qRT-PCR: M: TAKARA DL2000 marker; lane 1: Blank control (double distilled water); lane 2 and 3: WT and Tg F1 generation embryos at 3 dpf, respectively; lane 4: Positive control (plasmid). (D) Transgenic verification by westernblot: lane 1: WT embryo at 3 dpf; lane 2 and 3: Tg F1 and F2 generation embryos at 3 dpf, respectively. (E–F) EGFP (+) F0 mosaic zebrafish at 24 hours (×50) and 60 days post microinjection (×7.5). (G–H) EGFP (+) F1 Tg zebrafish at 24 hpf (×50) and 60 dpf (×10). (I) Expression of total *MYCN* (murine exogenous and zebrafish endogenous expression), which was increased gradually in Tg F1, F2 generation fish comparing with that in WT. (J) Expression of *NDRG1*, which is negative controlled by *MYCN* in human, was keeping a low lever in Tg F1 and F2 generation. **, P<0.01

About 60% of the embryos co-injected with the PSGH2/MYCN plasmid (Fig. 1A, B) and meganuclease exhibited EGFP (+) expression after heat shocked at 38°C for an hour. EGFP positive fish were screened under the fluorescent microscope on the next day and bred up to sex maturity (Fig. 1E–F), then crossed with the wild type (WT) fish. The Tg(*MYCN*:HSE:EGFP) F0 founders with the highest germline transmission rate were identified on the basis of fin genotyping (Fig. 1C–D) and EGFP expression of the F1 offspring after the same heat shock treatment (Fig. 1G–H). Eighteen of 256 (7.0%) mosaic F0 zebrafish were identified as the germline transgenic (Tg) zebrafish, including 8 males and 10 females. The Tg F1 generation were mated to create homozygous Tg(*MYCN*:HSE:EGFP) line. The EGFP (+) frequency of F2 offspring reached to 75% after heat shocked.

Using qRT-PCR, we have confirmed that expression of total *MYCN* (murine exogenous and zebrafish endogenous expression) was maintained at a high level in *MYCN* transgenic zebrafish from embryonic to adult (Fig. 1I), suggesting that the inducible system is not affected by development. N-Myc downstream regulated gene 1 (*NDRG1*) is negatively controlled by *MYCN* in human and defined as a differentiation related gene. We found that the expression of *NDRG1* in Tg fish was significantly under-expressed at 7 dpf and 60 dpf (Fig. 1J), implying that the MYCN/NDRG1 pathway is conserved between human and zebrafish.

### Massive immature hematopoietic cells emerged in blood circulation and infiltrated the organs of *MYCN* transgenic fish

**Figure 2:**
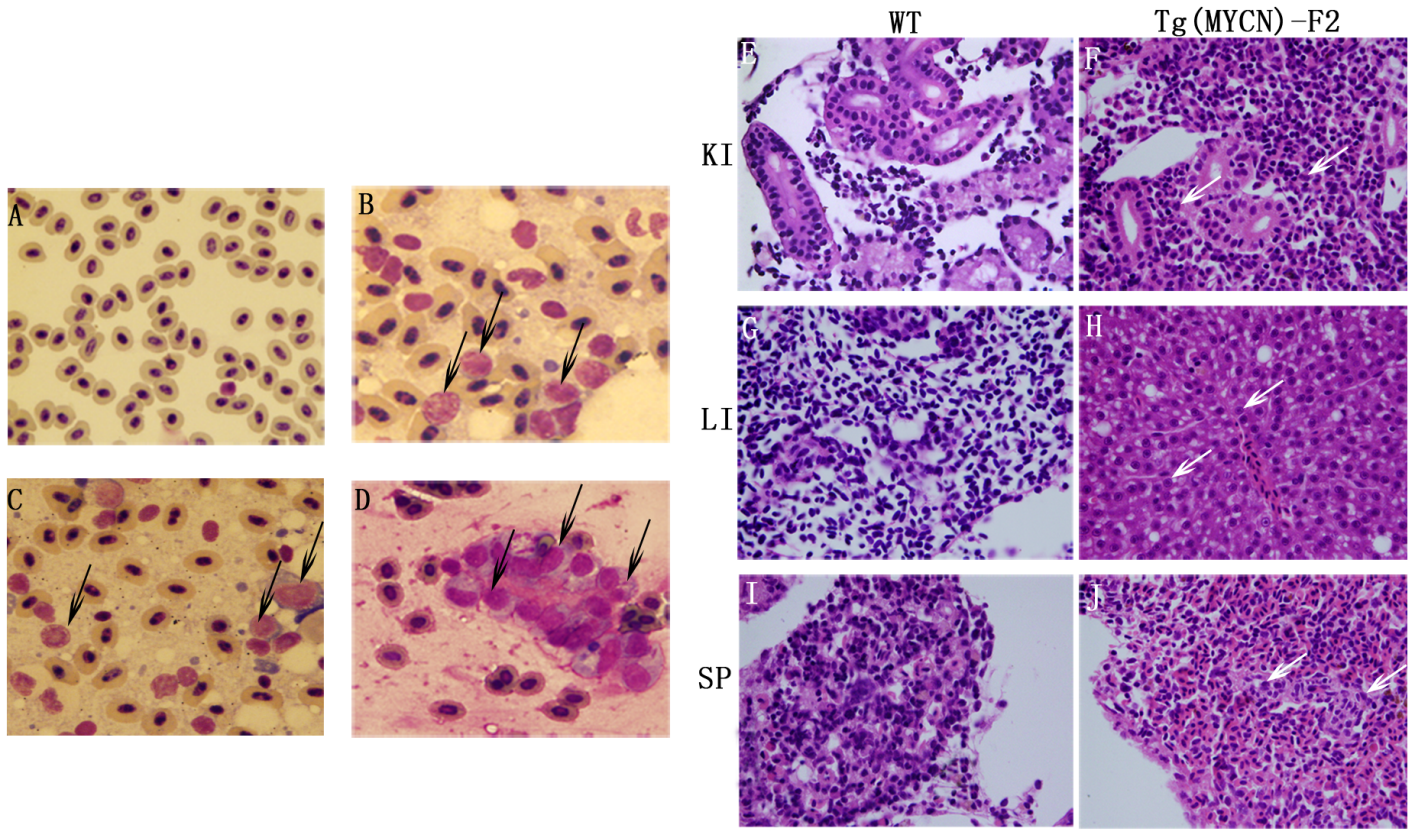
Cytological analysis of Tg(*MYCN*:HSE:EGFP) zebrafish.Cytology of hematopoietic cells from WT (A) and Tg(*MYCN*:HSE:EGFP) F0, F1, F2 generation (B, C, D) zebrafish at 60 dpf. The blood cells from WT fish were predominantly erythrocytes, with myeloid cells only occasionally observed. By contrast, erythrocytes were significantly inhibited in Tg fish, enriched for abundant blast-like cells, which are larger than the erythrocytes and have high nuclear to cytoplasmic ratios, containing multiple large nucleoli (black arrow). These blasts were similar to that of human AML peripheral blood. Transverse sections of kidney, liver, and spleen of WT (E, G and I) and Tg(*MYCN*:HSE:EGFP) F2 generation (F, H and J) zebrafish. Using HE staining, it showed that massive immature hematopoietic cells infiltrated in these organs of Tg fish (white arrow). KI, kidney; LI, liver; SP, spleen. (×1,000)

Using Wright Giemsa staining, we found that peripheral blood from both WT and Tg(*MYCN:*HSE:EGFP) zebrafish at 60 dpf contained a mixture of individual cells and clusters of cells, although cell clusters were more prevalent in samples from Tg fish than that from WT fish. The blood cells from WT were predominantly erythrocytes, with myeloid cells only occasionally observed (Fig. 2A). By contrast, the blood cells from the Tg fish contained abundant blast-like cells which were larger than the erythrocytes and had high nuclear to cytoplasmic ratios, containing multiple large nucleoli. These cells were similar to human AML blasts. Meanwhile, erythrocytes were significantly inhibited, whereas myeloid cells were increased (Fig. 2B–D).

**Figure 3:**
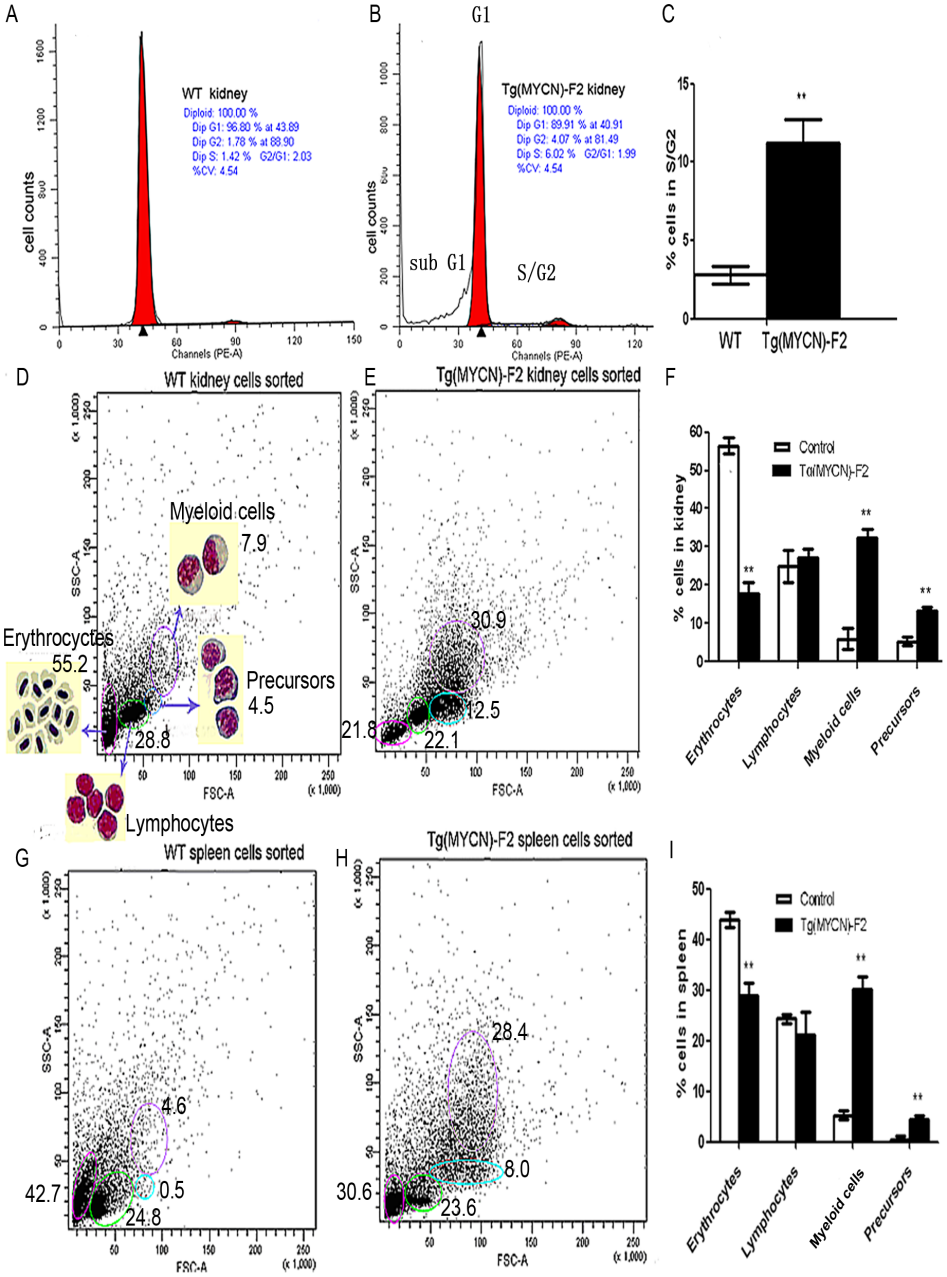
*MYCN* promoted cell cycle progression and increased the ratios of myeloid cells and its precursors. Single cell suspension from kidney of WT (A) and Tg(*MYCN*:HSE:EGFP) F2 generation (B) zebrafish was extracted at 60 dpf, treated with red blood cell lysis solution and stained with Propidium Iodide. Bars indicate the postion of modal DNA content peaks corresponding to the indicated G1 or S/G2 cell populations. It showed that *MYCN* increased the S/G2 ratio, histogram analysis in (C). In addition, elevated apoptosis was also detected in Tg samples as indicated by sub G1 peak (B). FACS analysed blood cells from kidney and spleen of WT (D, G) and Tg (E, H) fish at 60 dpf. Gated populations are as follows: erythrocytes, lymphocytes, myeloid cells, and blood cell precursors (morphologic feature was shown in D). All the detections repeated 3 times and analyzed in the two histograms (F, I) both indicated that myeloid cells and precursors were increased in Tg fish, correspondingly erythrocytes dramatically decreased, meeting the results of peripheral blood smear. **, P<0.01

With hematoxylin eosin (HE) staining, compared with WT (Fig. 3E, G, I), it showed that massive immature hematopoietic cells infiltrated the organs of adult Tg fish, such as the kidney, liver and spleen (Fig. 2F, H, J). These cells had high nuclear to cytoplasmic ratios, similar to the blast cells in the blood circulation.

### *MYCN* affects hematopoietic cell cycle and population distribution

Single cell suspension from kidney at 60 dpf was treated with red blood cell lysis solution and measured by flow cytometry to investigate the hematopoietic cell cycle and population distribution affected by *MYCN* expression. Increased S/G2 cell population in total detection living cells was observed in Tg fish (11.20±1.56) % compared to (2.40±1.13) % in WT fish (*p* < 0.01; Fig. 3A–C). In addition, elevated apoptosis (16.24±2.03) % was also detected in Tg samples as indicated by sub G1 peak (Fig. 3B).

Next, the lineages of hematopoietic cells from kidney and spleen were investigated by fluorescence-activated cell sorting (FACS) at 60 dpf. In the kidney, the erythrocytes population was inhibited (56.50±2.12 *versus* 17.90±2.97), while myeloid cells (5.95±2.76 *versus* 32.45±2.19) and precursors (5.25±1.06 *versus* 13.25±1.06) were increased in Tg fish. However, lymphocytes ratio exhibited no difference between the two groups (Fig. 3D–F). The changes in the spleen, similar to those in the kidney, showed that erythrocytes were inhibited (43.90±1.56 *versus* 28.80±2.55), while myeloid cells (5.20±0.85 *versus* 30.20±2.55) and precursors (0.50±0.71 *versus* 4.50±0.71) were increased in Tg fish (Fig. 3G–I). These results coincided with the findings of peripheral blood smears and tissue sections.

### *MYCN* regulates lineage-specific hematopoietic transcription factors in hematopoiesis

**Figure 4:**
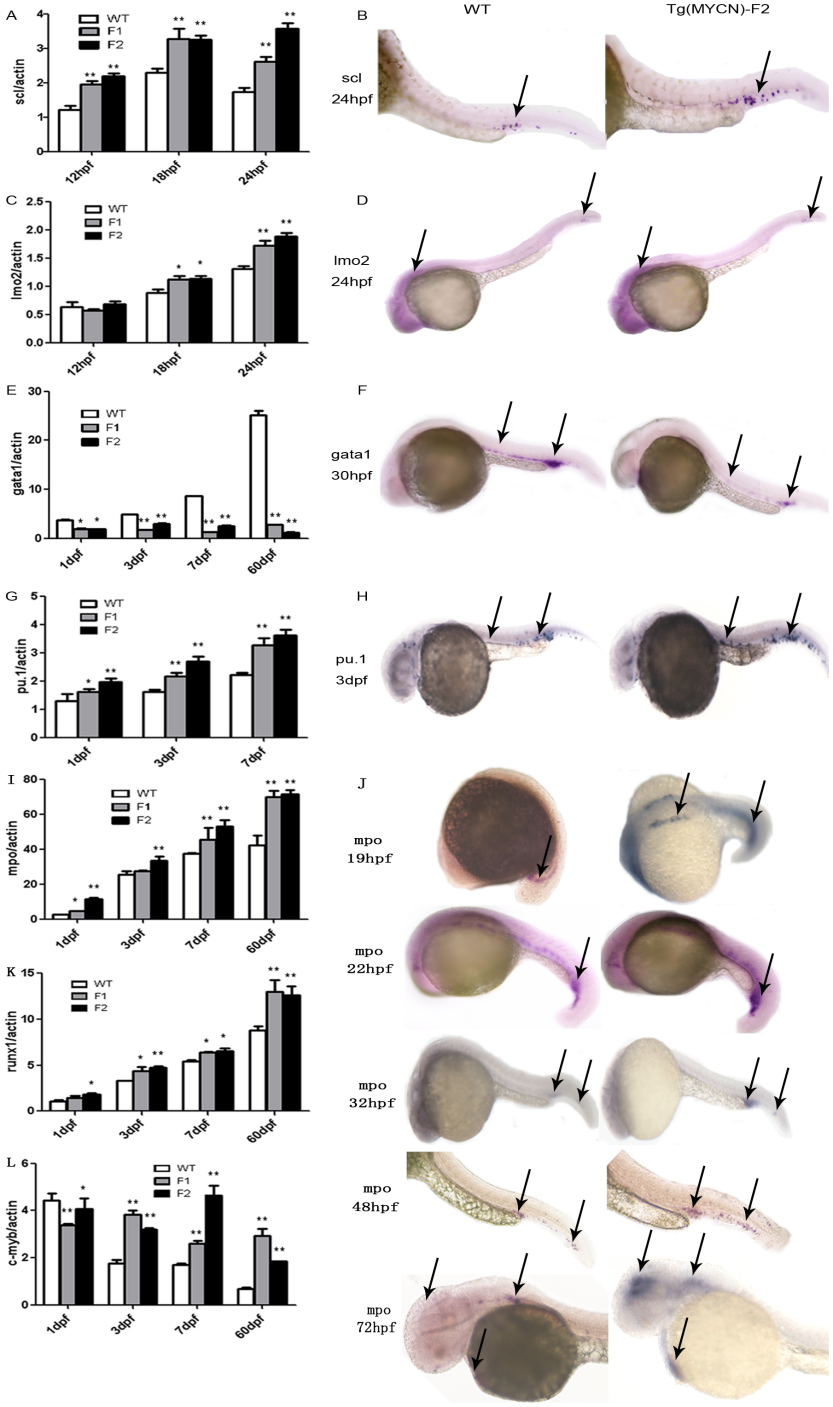
*MYCN* reprogrammed hematopoiesis by regulating lineage-specific hematopoietic transcription factors. *Scl* (A) and *lmo2* (C) expressed in WT, and Tg(*MYCN*:*HSE*:*EGFP*) F1, F2 generation embryos at 12 hpf, 18 hpf and 24 hpf. *In situ* hybridization of *scl* (B) and *lmo2* (D) at 24 hpf. *MYCN* increased the two factors expression. *Gata1* (E), *pu.1* (G) and *mpo* (I) were detected by RQ-PCR in WT, Tg F1 and F2 embryos at 1 dpf, 3 dpf, 7 dpf and 60 dpf. *In situ* hybridization of *gata1* (F), *pu.1* (H) and *mpo* (J) in WT and Tg F2 embryos indicated that *gata1* was downregulated, however, *pu.1* and *mpo* were increased in Tg group, meaning that myelopoiesis was promoted while erythropoiesis was inhibited. *Runx1* (K) and *c-myb* (L) expression were detected in WT and Tg F1, F2 generation zebrafish at 1 dpf, 3 dpf, 7 dpf and 60 dpf. *Runx1*, involved in definite hematopoiesis regulation, was upregulated in Tg group. *C-myb*, predominantly expressed in immature hematopoietic cells, were higher expression in Tg group, which suggests that *MYCN* overexpression results in accumulation of immature hematopoietic cells in adult fish. *, P<0.05; **, P<0.01

We sought to understand how *MYCN* exerts the observed hematopoietic phenotype. The primitive HSCs are produced intraembryonically in ventral mesoderm derived tissue called the intermediate cell mass (ICM). During this wave, the anterior lateral mesoderm (ALM) of the embryo generates myeloid cells, while the posterior lateral mesoderm (PLM) generates mostly erythrocytes and some myeloid cells. Stem cells transcription factor (*scl*) is required in the promotion of primitive hematopoiesis [11]. Its expression was measured by qRT-PCR in WT and Tg F1, F2 generation embryos at 12 hpf (1.212±0.207 *versus* 1.958±0.180, 2.189±0.152), 18 hpf (2.301±0.198 *versus* 3.275±0.518, 3.249±0.203) and 24 hpf (1.738±0.200 *versus* 2.618±0.229, 3.572±0.263) (*p*<0.01, Fig. 4A). *In situ* hybridization, *scl* was also showed higher expression in Tg embryo than its WT counterpart (Fig. 4B). LIM only protein 2 (*lmo2*), which is expressed in hematopoietic progenitors, acts in parallel with *scl* as an important hematopoietic regulator. In the embryos of *MYCN* transgenic zebrafish, *lmo2* was slightly upregulated compared to WT counterpart (*p*<0.05, Fig. 4C–D). *Gata1* is a master regulator in erythrocyte development. In zebrafish, *gata1* is expressed from the 5 somite stage in the PLM. We found that the expression levels of *gata1* significantly downregulated in Tg embryos. Its expression was measured by qRT-PCR in WT and Tg F1, F2 generation at 1 dpf, 3 dpf (4.89±0.15 *versus* 1.68±0.07, 2.99±0.08), 7 dpf and 60 dpf (25.21±1.36 *versus* 2.75±0.08, 1.22±0.03) (Fig. 4E–F). *Pu.1* is a master regulator of myeloid cell development. It is expressed from the 6 somite stage in the ALM. *Pu.1* was upregulated in the embryos of F1 and F2 generations of Tg (Fig. 4G–H). Its expression was detected in WT and Tg F1, F2 generation at 1 dpf, 3 dpf (1.62±0.15 *versus* 2.17±0.19, 2.69±0.29) and 7 dpf (2.21±0.15 *versus* 3.26±0.44, 3.62±0.36). It has been reported that in *gata1* knockdown embryos, blood cells in the ICM switched their fates to myeloid cells in the presence of *pu.1,* suggesting an interplay between *gata1* and *pu.1* during primitive hematopoiesis to balance erythroid and myeloid populations [12]. So we supposed that *MYCN* could promote primitive hematopoiesis by upregulating *scl* and *lmo2* expression, and promote myelopoiesis by inhibiting *gata1* expression.

From 24 hpf, these primitive blood cells start to circulate throughout the embryo. Subsequently, the definitive HSCs emerge from the ventral wall of the dorsal aorta. *Runx1*, a member of the runt family of transcription factors, demonstrated to be required in definitive hematopoiesis. In our experiment, we observed that the expression of *runx1* was slightly upregulated in Tg zebrafish (Fig. 4K). In addition, we also measured the expression of Myeloperoxidase (*mpo*), the granulocyte specific gene and considered as the symbol of mature neutrophils, whose expression was first detected in between 18 and 20 hpf with the distribution from ICM to rostral blood island (RBI). In Tg fish, the *mpo* expression was upregulated (Fig. 4I–J), a finding that is consistent with the increased expression of *pu.1* (Fig. 4G–H). In addition, the expression level of *c-myb*, whose expression is predominantly present in immature hematopoietic cells and decreases during cell differentiation, did not decrease with cell growth and differentiation in Tg fish (Fig. 4L). It suggested that a large number of immature blood cells accumulated in blood circulation.

### Transcriptional changes in the blood of *MCYN* transgenic embryos

Using Agilent microarray analysis, we obtained a total of 626 differentially expressed genes (DEGs) in the blood cells of Tg(*MYCN*:HSE:EGFP) F1 generation *versus* WT embryo at 3dpf. There were 342 genes upregulated and 284 genes downregulated (>2-fold change in expression, P<0.01). The number was too large to pinpoint the crucial DEGs. As a transcription factor, *MYCN* can directly or indirectly alter several downstream pathways. NIH-DAVID software was used to perform the functional analysis of the DEGs. Several Kyoto Encyclopedia of Genes and Genomes (KEGG) pathways were significantly enriched (False Discovery Rate, P-value <0.01, Benjamini <0.05) (Table 1). Some were upregulated in the Tg fish, including cell cycle, glycolysis/gluconeogenesis, fatty acid metabolism, MAPK, tyrosine metabolism and *p53* signaling pathway. Meanwhile, mismatch repair, homologous recombination, base excision repair and transforming growth factor β (TGFβ) were downregulated. Majority of the above alterations in signaling pathways were associated with human hematopoietic disorders and malignant transformation of blood cells [13], [14], [15], [16], [17], [18].

**Table 1:** Changes in Tg(MYCN:HSE:EGFP) zebrafish hematopoietic cells.

Term	regulate	Count^*^	%^#^	P-Value	Benjamini
Cell cycle	+	42	3.3	4.5E-17	2.3E-15
Glycolysis/Gluconeogenesis	+	20	1.2	3.7E-07	0.000044
Tryptophan metabolism	+	14	0.8	8.6E-06	0.0005
Fatty acid metabolism	+	13	0.8	9.4E-06	0.00037
Cardiac muscle contraction	+	20	1.2	0.000038	0.0011
Biosynthesis of unsaturated fatty acids	+	9	0.5	0.000057	0.0013
Phenylalanine metabolism	+	9	0.5	0.00014	0.0027
Tyrosine metabolism	+	11	0.7	0.0004	0.0066
MAPK	+	11	0.7	0.001	0.015
P53 signaling pathway	+	13	1	0.0021	0.02
Fatty acid elongation in mitochondria	+	6	0.4	0.0022	0.028
Arginine and proline metabolism	+	13	0.8	0.0038	0.043
Valine, leucine and isoleucine degradation	+	10	0.6	0.0069	0.071
Alanine, aspartate and glutamate metabolism	+	9	0.5	0.0069	0.066
Butanoate metabolism	+	8	0.5	0.008	0.07
DNA replication	-	24	1.9	1.1E-17	1.1E-15
Mismatch repair	-	14	1.1	5.9E-10	2.1E-08
Homologous recombination	-	12	0.9	9.7E-07	0.000025
Base excision repair	-	13	1	1.9E-06	0.000039
Spliceosome	-	23	1.8	0.000033	0.00058
Nucleotide excision repair	-	13	1	0.000038	0.00056
Pyrimidine metabolism	-	19	1.5	0.000056	0.00072
transforming growth factor β	-	21	1.6	0.00063	0.0073
One carbon pool by folate	-	6	0.5	0.0021	0.022

+: upregulated; -: downregulated. *: genes involved in the term. #: involved genes/total genes. P-Value: the threshold of EASE Score, a modified Fisher Exact P-Value, for gene-enrichment analysis (≤0.01). Benjamini: Benjamini and Hochberg's false discovery rate, <0.05.

**Figure 5:**
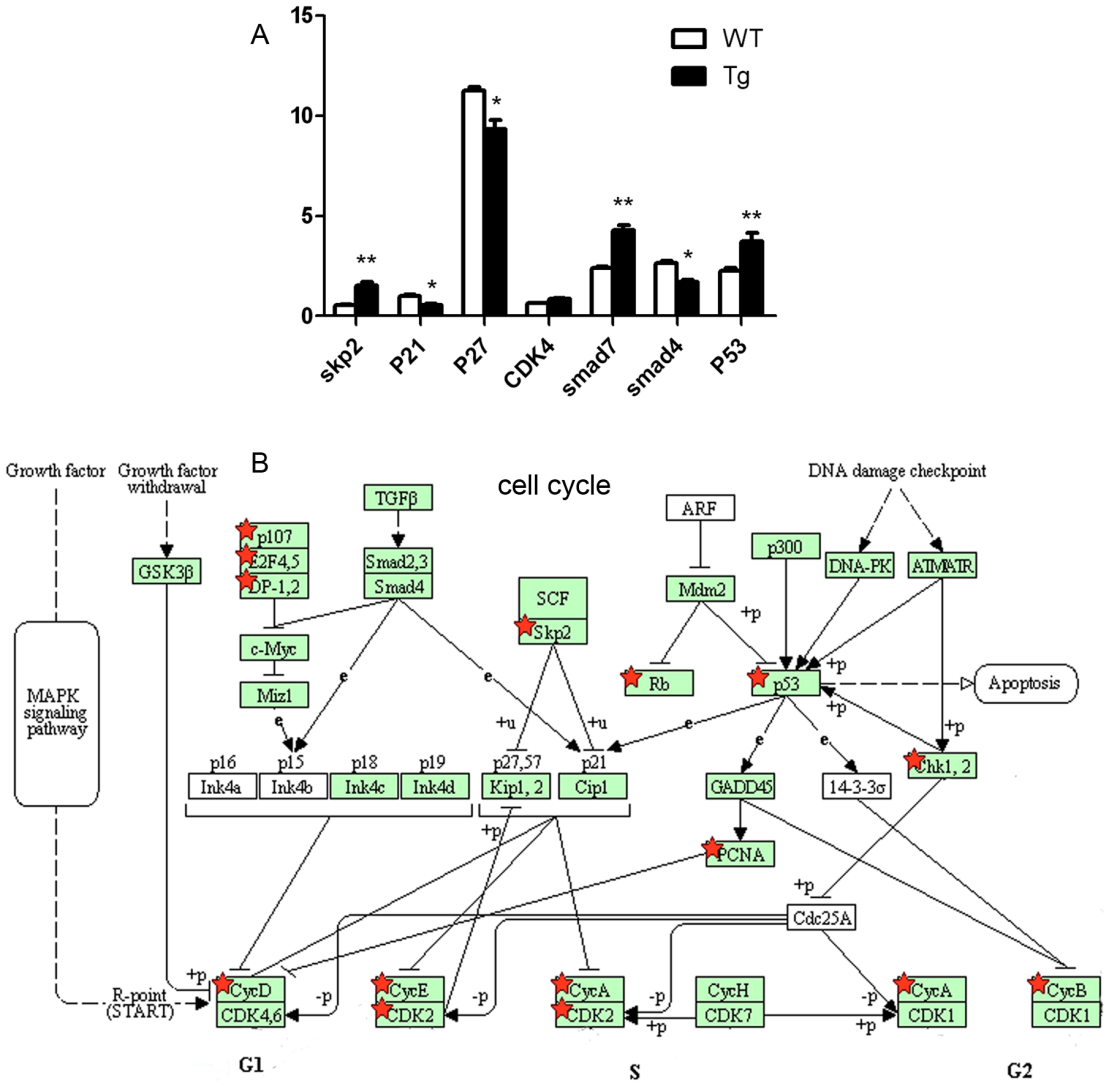
DEGs in Tg(*MYCN*:*HSE*:*EGFP*). (A) The expression of *skp2*, *p53* and *smad7* were upregulated, while the expression of *p21*, *p27* and *smad4* were downregulated in Tg embryos. CDK4 exhibited no difference between WT and Tg groups. *, P<0.05; **, P<0.01. (B) The cell cycle pathways were analyzed and summarized by NIH-DAVID software. The upregulated genes were marked with red star in above figure.

The detail visualization of cell cycle progression allowed uncovering upregulated genes in *MYCN* transgenic fish (Fig 5A). S-phase associated kinase 2 (*Skp2*) emerged a higher expression of in Tg embryos. *Skp2* forms a part of the SKP1-Cul1-Fbox (*SCF*) complex, which executes the degradation of negative regulators of the cell cycle such as p27^kip1^, p57^kip2^ and p21^cip1^. Sequentially, the transcriptional repression to cyclin A, E and other E2F target genes was weakened. In addition, MAPK signaling pathway was highly activated through upregulating the expression of *FGF*, *PDGF*, *BDNF*, *CACN*, *Ras* and *MKP*, which directly promoting the expression of cyclin D (detail not shown). The TGFβ signaling was inhibited by the upregulation of *Smad 6/7* and *IFN γ* in Tg fish (detail not shown). Taken together, these changes contributed to promote cell cycle progression. Activation of p53 pathway was also identified with upregulation of checkpoint kinase-1 (*Chk1*) and *p53* in *MYCN* transgenic fish. *Chk1* is a serine/threonine kinase that is involved in the response to single strand DNA breaks and the induction of *p53* expression. High levels of *p53* expression in the Tg zebrafish line resulted in the promotion of two biological functions: apoptosis (*Bid* gene) and antioxidant activity (*B99* gene) (detail not shown). The promoter region of *p53* contains a non-canonical E-box. Recently, ChIP on chip has identified that *MYCN* directly binds to the non-canonical E-box in *p53* and is a direct transcriptional target gene of *MYCN* in neuroblastoma [19]. Hence, the activation of *p53* expression by *MYCN* expression in Tg fish may reveal an important mechanism by which *MYCN* sensitizes cells to increased apoptosis.

Furthermore, we measured the expression of the main factors involved in these pathways by qRT-PCR. It showed that the expression of *skp2*, *p53* and *smad7* were upregulated, while the expression of *p21*, *p27* and *smad4* were downregulated (Fig 5A), which consisted with these microarray results.

### Followed-up of the transgenic zebrafish

**Figure 6:**
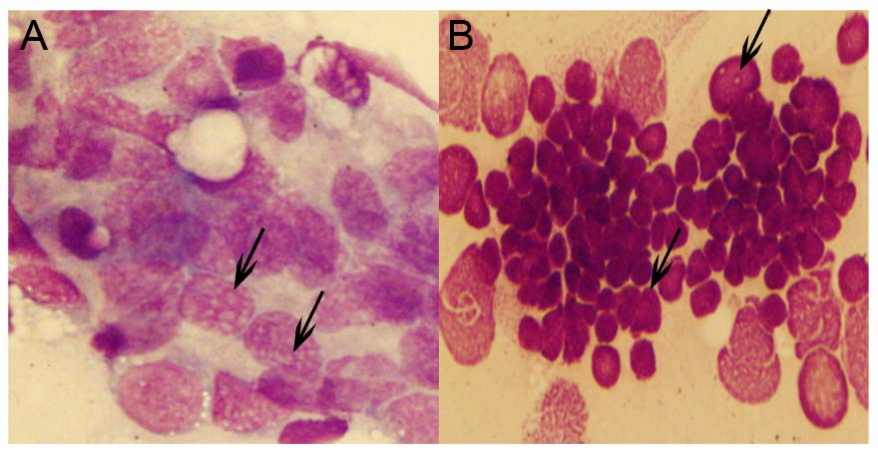
The examination of transgenic zebrafish persistency. (A) The peripheral blood cells in Tg(*MYCN*:HSE:EGFP) F1 generation showed no difference in between 2-month and 8-month pf in the amount of cells containing multiple nucleoli. (B)The peripheral blood cells in Tg fish at 13 months pf, after losing the EGFP expression, showed clusters of stripped nucleus.

Different from 3 years lifetime of the WT zebrafish, all the F0 founders died within 5∼13months (mean: 9.5 months). Most of the F1 and F2 generation older than one year lost the ability of fecundity. Gradually loss of the EGFP expression and began to die about the age of 10 months. We got the similar results of the peripheral blood cells smears in Tg fish at 8 mouths pf to that in 2 months pf (Fig. 6A). When the EGFP expression vanished, the peripheral blood appeared lots of clusters of stripped nucleus (Fig. 6B). These results suggested that heat-shock efficacy can maintain for 10 months and be suitable for doing research in the early period. A small amount of sick fish can partially self-recovered.

## Discussion

We report here a *MYCN* transgenic zebrafish model with a phenotype that recapitulates main aspects of human AML such as distorted proliferation, metabolic disturbance, increased myeloid cells and their precursors accumulation in peripheral circulation, spleen and kidney marrow, suggesting that *MYCN* plays a role in the etiology of AML. More importantly, zebrafish offers the advantage of high-throughput scale in the study of *MYCN* function *in vivo*. Using this *MYCN* stable-expression model enables us to track the molecular alterations that occur well before the appearance of morphological phenotypes, and to determine the roles of candidate *MYCN* target genes. We demonstrated that *MYCN* reprograms hematopoietic cell fate by regulating *NDRG1* and several lineage-specific hematopoietic transcription factors *in vivo*.

A number of evidences accumulated showing that the Myc protein (encoded by the main members of *Myc* family—*MYCC* and *MYCN*) plays a major role in hematopoiesis and hematologic malignancies [20]. *MYCC* or *MYCN* transcripts are co-expressed at similar levels in long-term HSCs (LT-HSCs). Their activity is emerging as a key element in acquisition and maintenance of stem cell properties, and either of them alone could promote HSCs proliferation [21]. Enforced *MYCC* expression in HSCs results in reduced self-renewal activity and increased proliferation of the Myc-expressing HSCs by down-regulating the expression of p27^kip^ [22]. Our study showed that overexpression of *MYCN* also downregulated p27^kip^ and p21^cip1^ expression in hematopoietic cells (Fig. 5A), accompanied by up-regulation of early transcription factors, including *scl, lmo2*, and *runx1* (Fig. 4A–D, 4K). Knockdown of *scl* in zebrafish leads to complete loss of primitive hematopoiesis [11], loss of *c-myb* and *runx1* expression in the dorsal aorta [23], and severely disrupted endothelial differentiation in HSC formation [24]. El Omari K reported that *lmo2* functions as the scaffold for a DNA-binding transcription regulator complex, on condition knockdown of *lmo2* leads to complete loss of PLM primitive hematopoiesis [25]. Using transgenic mice carrying human CD34 PAC gene, Levantini identified a novel downstream regulatory element (DRE) that is bound by *runx1* and is necessary for human CD34 in long-term (LT)-HSCs [26]. Recently, Herbomel confirmed that zebrafish HSCs emerge directly from the aortic floor and this process is dependent on *Runx1* expression [27]. Taken together, our findings suggested an important role of *MYCN* in HSC proliferation and survival.

Taking the advantage of transparence in embryo, we performed whole-mount *in situ* hybridization and found that the expression of *gata1* was downregulated while the expression of *pu.1* and *mpo* was upregulated in *MYCN*-overexpressing zebrafish embryos (Fig. 4E–J). Acosta and colleagues have reported that the expression of *MYCC* blocks p27-mediated erythroid differentiation by impairing the upregulation of many erythroid-specific genes, including *NFE2*, *JUNB*, and *gata1* [28], a similar effect was also observed in our *MYCN* Tg model (Fig. 5). There is a cross-inhibitory mechanism between the expression of *gata1* and *pu.1* [12], indicating that the level of *pu.1* expression is determined by the ability of *MYCN* to regulate both *gata1* and *pu.1*, which leads to increased the repopulation of myeloid cells. However, what are the reasons for the long-lasting blockade of maturation of myeloid cells? Generally, *MYCN* transcripts progressively decrease during the initial differentiation step into short-term HSCs (ST-HSCs) to ensure the expression of *NDRG1*. Tschan reported that significantly higher levels of *NDRG1* mRNA were detected in granulocytes of healthy donors than in primary AML cells, moreover, silencing of *NDRG1* diminished neutrophil differentiation of leukemic cell lines [29]. Along with our data, these findings suggest that there is an association of low *NDRG1* levels with an immature cell phenotype (Fig. 1J, 2B–D). Furthermore, *lmo2* has been proved as a downstream target of many oncogenes leading to immortalize hematopoietic progenitors [30], [31].

Several reports illustrated, driven by specific promoters, that overexpression of *MYCN* or *MYCC* in mice or zebrafish causes lymphoma or leukemia. Transgenic mice overexpressing *MYCN* and *MYCC Via* the Eµ enhancer (targeted to B cell) develop lymphoma after a latency period of 2 to 5 months [32]. Using rag2 promoter (target to immature T-cell), Langenau generated *MYCC*-induced T cell acute lymphoblastic leukemia in zebrafish [33]. In addition, bone marrow retrovirally transduced with *MYCN* developed monoclonal AML in mice, while *MYCC* retrovirus was not leukemogenic in the same system [4]. In this model, microarray analysis revealed decreased TGFβ signaling (up-regulation of *Smad7* and down-regulation of *TGFβ*) and increased c-Jun-NH2-kinase (JNK) signaling in *MYCN*-overexpressing cells. Using methylcellulose-based culture (MC) serial replating assay, *MYCN* stimulated the colony-forming activity of myeloid progenitors. All recipients of bone marrow transduced with retrovirus bearing *MYCN* gene developed myeloid disease and died within 50 days after transplantation. All sick mice showed a markedly enlarged spleen and liver, increased numbers of peripheral WBC, mild anemia and almost consisted exclusively of myeloid blasts in the bone marrow (90.2±3.1%, n = 5). Therefore, *MYCN* overexpression is highly oncogenic in mouse myeloid cells.

Driven by HSP, our *MYCN* transgenic zebrafish also showed increased numbers of peripheral myeloid cells, anemia and vast myeloid blasts infiltrated in spleen, liver and kidney (Fig 2, 3). However, the transgenic zebrafish showed lower percentage of blasts in kidney and a longer lifespan than those in transgenic mice. These discrepancies may result from the species differences and the leakiness of the promoter in the generation of transgenic lines. By microarray analysis, we also found that *MYCN* decreased TGFβ signaling (table 1). In addition, pathways involved in cell cycle progression, glycolysis/gluconeogenesis, fatty acid metabolism, MAPK/Ras, tyrosine metabolism and p53 signaling were enhanced, while those of mismatch repair, homologous recombination and base excision repair were inhibited. Rapidly proliferating tumors are often dependent on glycolysis for ATP production even in normoxia, what is defined as the Warburg effect. Akers and colleague reported that all acute leukemia subtypes (pre-B ALL, T-ALL and AML) demonstrated growth arrest and cell death when treated the novel glycolysis inhibitor 3-BrOP [34], indicating that Warburg effect also exists in AML. Genes that correlated with glycolysis/gluconeogenesis were significantly upregulated in our experiment (n = 20, P = 3.7E-07). Since the samples for microarray analysis were collected more than 2 days after heat shock, some alteration may result from an indirect effect of *MCYN* expression. These data requires further experimental verification.

Work by Kawagoe demonstrated that the expression of Cdk4 and the percentage of cells in S/G2-M phase were significantly elevated in cell cultures overexpressing *MYCN* [4]. In our *in vivo* experiment, we found that *skp2* up-regulation mediated the suppression of the p27^kip1^, p57^kip2^ and p21^cip1^, which in turn enhanced the expression of cyclin A, D, E and CDK2. The expression of CDK4 that measured by qRT-PCR and microarray analysis exhibited no difference between WT and Tg groups (Fig 5). The discrepancy may result from the condition of experiments (*in vivo versus in vitro*). Previous finding reported that *skp2* expression was decreased by 2-fold in the absence of *MYCN* expression [35]. Another study showed that p27^kip1^ level in *MYCN*-overexpressing cells was restored by *skp2* knock down [36], indicating that the down-regulation of p27^kip1^ by *MYCN* was mediated by the expression of *skp2*. *P21* was another negative regulator of the cell cycle which was induced by p53 and suppressed by *skp2*. It was ultimately downregulated and lead to a failure of G1 arrest in our transgenic zebrafish (Fig 5). Our data is supported by a previous finding that *MYCN* amplification attenuated p21^WAF1^ induction and failed to induce G1 arrest after DNA damage [19].

HSP has no tissue-specific preference, yet heat stress exhibits more direct and far-reaching influence on white blood cells than other cells (such as neurocytes). Moreover, *MYCN* overexpression is highly oncogenic in myeloid cells. Thus the establishment of *MYCN* transgenic zebrafish with the uniform phenotype of the tumor cells shows better resemblance of the feature of human AML.

AML is the most common hematological malignancy in human adults. Understanding the molecular mechanisms and complex transcriptional networks by which *MYCN* exerts its influence on hematopoietic progenitor cells will shed light on developing targeted therapeutic strategies. This model will provide a useful tool to conduct whole-organism chemical suppressor screens to identify compounds that can reverse *MYCN* function *in vivo*, for example, Skp2 inhibitors and NDRG1 activators.

## Materials and Methods

### Ethics statement

This work was approved by the Institutional Animal Care and Use Committee (IACUC) of Shanghai Research Center for Model Organisms (Shanghai, China) with approval ID 2010-0010.

### Construction of PSGH2/MYCN plasmid

A mouse-*MYCN* fragment was extracted from HA-MYCN plasmid [4] and subcloned into the EcoRI and EcoRV (Takara, Japan) sites of the PSGH2 vector [37] to obtain the m*MYCN*-HSE-EGFP construct (Table 2, Fig. 1A). The PSGH2 vector contains eight HSE sequence (AGAACGTTCTAGAAC) [38] allows the symmetrical addition of a CMV minimal promoter to both ends in order to drive the expression of the genes of interest (one side is EGFP and the other side is *MYCN*) flanked by 5V and 3V globin UTRs and SV40 polyadenylation (pA) signal (I-SceI meganuclease recognition sites) (Fig. 1B).

**Table 2:** Primers used for RT-PCR.

Name	Forward Primer	Reverse Primer
clone *mMYCN*	TAT*GAATTCA*TGCCCAGCTGCACCGCGTCCAC (EcoRI site in italics)	GCC*GATATCT*TAGCAAGTCCGAGCGTGTTCGATC (EcoRV site in italics)
*scl*	AGCCATAAGGTGCAGACCAC	CGTTGAGGAGCTTAGCCAGA
*lmo2*	TCTTTCTGAAGGCCATCGAGCAGT	GCACAGCTTTCTGCCGAGTTTGTA
*runx1*	TTGGGACGCCAAATACGAACC	ATATCACCAAGGGCAACCACC
*gata1*	ATGAACCTTTCTACTCAAGCT	GCTGCTTCCACTTCCACTCAT
*pu*.1	GTTCCTGCTTGACCTTCTGCGAAA	TCAGTGCTCTTGCCATCTTCTGGT
*mpo*	GCTGCTGTTGTGCTCTTTCA	TTGAGTGACAGGTTTTGG
*c-myb*	GCTGACTAGCTCTGTGCTGATG	GCTGAGGTATTTGTGCGTGG
*NDRG1*	CCCCAGGACAGCAAGAAGG	GCAGGGTAATCCAAAGCAAAC
m-*MYCN*	GTGTCTGTTCCAGCTACTGCC	TCATCTGAGTCGCTCAAGGTATC
z-*MYCN*	AAGGCAGCAAAGGTGGTCAT	GAGCGTAAAGGGTTAGCGAGT
*P21*	GAACGATGTGCTGCACTCCC	TGTCAATAACGCTGCTACGAGAC
*P27*	CGACTGTAGGGTAACGGAGCA	GGGTGTCGGACTCAATGGTT
*P53*	CGGCGATCATGGATTTAGGC	TTCAGCCACATGCTCGGACT
*skp2*	ATCTGGGACTGAGCCGTTGT	GAGAACGGCTGCGTGTTGAT
*cdk4*	TGCCGAGATGTTCAGACGC	GCTGAAGTTGTGGTGGGAAAGA
*smad7*	GGTTCTGTGCCTGCTTCCA	TGCCCTGAGGTAGGTCGTAGA
*smad4*	AGACCTCCACATACCACCACA	GTCCATCTCGAAGTAGGCAAT
*cdk4-2*	AACATCGTCAGGCTGATGGAC	TCCCGATGGAGAACTCGATTAG
β-actin	CCTGACCGAGAGAGGCTACA	CGCAAGATTCCATACCCAAG

### Microinjection of PSGH2/MYCN plasmid into AB embryos and Generation of the Tg(MYCN:HSE:EGFP) zebrafish line

Zebrafishes were maintained as described by Westerfield [39]. Developmental stages refer to hpf or dpf. Fertilized wild-type AB fish eggs were microinjected through the chorion into the cytoplasm at the one-cell stage of development. The PSGH2/MYCN plasmid was co-injected with I-SceI meganuclease enzyme (0.5units/µL) in 1 µl of I-SceI buffer (New England Bio Labs). A pressure injector (IM-300, NARISHIGE) was used with borosilicate glass capillaries. After injection, the embryos were collected in Petri dishes and incubated at 28°C. They were heat shocked at 38°C for 1 hour once between 14 to 18 hpf to induce the EGFP expression and *MYCN* phenotypes. EGFP positive fish were screened under the fluorescent microscope on the next day and bred up to sex maturity, then crossed with the wild type (WT) fish. The transgenetic offspring also need to be heat shocked for an hour to induce EGFP and *MYCN* expression.

### Real-time quantitative reverse transcription PCR (qRT-PCR)

qRT-PCR was performed as described [40]. Briefly, total RNA was extracted with the RNeasy kit (Qiagen, Japan) and treated with DNase I (Promega, Japan). cDNAs were synthesized from 1 µg of total RNA using Quantscript RT Kit (TIANGEN, China). Real-time RT-PCR was performed using 400 ng of cDNA templates in an ABI StepOnePlus System (Applied Biosystems, USA). PCR primers were designed to span introns and listed in Table 2. Measured cycle threshold (Ct) values represent log2 expression levels. Each target gene was calculated using the 2^−ΔΔCT^ method [41].

### In situ hybridization

Whole-mount *in situ* hybridization was performed using digoxigenin-labeled antisense riboprobes for hematopoietic transcription factors (*scl*, *lmo2, gata1*, *pu.1, mpo*) as previously described [42].

### Cytological analysis

After transferred into ice water for 30 sec, blood was harvested from zebrafish by making a lateral incision just posterior to the dorsal fin in the region of the dorsal aorta. Blood was rapidly collected by a micropipette tip and used in preparing blood smears [43]. Slides were then stained with Wright Giemsa stain and examined under oil immersion by light microscopy. Identification of zebrafish peripheral blood cells was based, in part, on previous descriptions of teleost blood cells [44].

### Organs dissection and histological analysis

Dissection of organs from adult zebrafish was performed as previously described [45]. These organs were used for fluorescence activated cell sorting (FACS) and tissue sections. Paraffin-embedded tissue sections of kidney, liver and spleen were stained with H&E to confirm the morphological changes.

### Flow cytometric analysis and cell sorting

Kidney and spleen isolated from adult zebrafish were homogenized in ice-cold 0.9× phosphate-buffered saline (PBS) containing 5% fetal bovine serum, and then passed through a 40 µm filter to obtain a single cell suspension. Cells were washed once with the same solution, stained with Propidium Iodide (Sigma-Aldrich) at a final concentration of 1 µg/mL and analyzed by fluorescence-activated cell sorting (BD FACS ARIA II SORP, USA) [46]. Cell size was represented by forward scatter (FSC), and granularity was represented by side scatter (SSC). Gated populations were as follows: erythrocytes, lymphocytes, myeloid cells, and blood cell precursors. All the detections repeated 3 times. Populations of cells within each gate were described as mean percentages of total cells (mean±SD, %) and shown in histogram.

### Microarray analysis

The WT and Tg(*MYCN*:HSE:EGFP) F1 generation embryos were heated shocked at 38°C for 1 hour at 16 hpf and hematopoietic cells were collected at 3 dpf by flow cytometry. Total RNA from 5×10^4^ cells was isolated with Trizol (Invitrogen). The samples were processed and subsequently analyzed in triplicate on Zebrafish Oligo Microarrays (Agilent Technologies Italia, Italy) which contain 43,554 sets of probes. The microarrays were scanned in an Agilent DNA Microarray Scanner and the images were processed using Feature Extraction software. Functional annotation analysis was performed using NIH-DAVID software (version 6.7) [47]. Our aim was to find the most relevant Gene Ontology (GO) terms associated with DE genes. For this purpose, the significance p-value threshold was set <0.01, with Bonferroni multiple testing correction (<0.05).

### Statistical Analysis

Data were reported as the means±S.E. and statistically compared using one-way analysis of variance followed with least significant difference or Student's *t* test. Statistical significance was accepted when *p*<0.05. All statistic analyses were carried out in GraphPad Prism 5.
